# Hypertension in non-obese children and BMI in adulthood: the Bogalusa heart study

**DOI:** 10.1186/s12872-023-03699-6

**Published:** 2024-01-03

**Authors:** Lingli Zhao, Hua Qu, Jiahui Ouyang, Yanyan Meng, Zhuye Gao

**Affiliations:** 1 Xiyuan Hospital, China Academy of Chinese Medical Sciences, No.1 Xiyuan Playground, Haidian District, Beijing, 100091 China; 2 National Clinical Research Center for Chinese Medicine Cardiology, Beijing, 100091 China; 3 Beijing University of Chinese Medicine, Beijing, 100029 China

**Keywords:** Hypertension, Body mass index, Regression analysis, Low-density lipoprotein cholesterol, Risk factors, Database, Gut microbiota, Inflammation

## Abstract

**Objective:**

This study explored the association between hypertension(HTN) in non-obese children body mass index (BMI) in adulthood.

**Methods:**

A retrospective analysis of 1111 participants from the Bogalusa Heart Study was conducted, in which data on hypertension history during childhood in non-obese children, anthropometric and cardiovascular risk factors and other indicators from cross-sectional examinations in adulthood were collected. BMI was used as both a continuous and a categorical variable, and multivariate linear regression modelling and logistic regression modelling were used.

**Results:**

Of the 1111 participants finally enrolled, 40 (3.60%) had HTN during childhood. After adjusting for demographic characteristics, lipid, glucose and insulin levels in childhood, and smoking status, alcohol intake, and disease history as adults, HTN among non-obese children was positively associated with BMI in adulthood (β = 2.64 kg/m^2^, 95% CI: 0.88–4.40, *P* = 0.0033), and the odds of being overweight or obese was 3.71 times higher in the group with a history of hypertension in childhood than those without a history of HTN(95% CI: 1.11–12.46, *P* = 0.0337).

**Conclusion:**

Among non-obese children, hypertension is at risk for higher levels of BMI in adulthood. Identifying and controlling blood pressure and childhood may aid in the prevention of adult obesity.

## Background

Body mass index (BMI) is frequently used to assess obesity in humans, with higher BMI values associated with health risks and mortality [1, 2]. In adults, the World Health Organization defines a BMI ranging from 25-to < 30 kg/m^2^ as overweight and a BMI ≥ 30 kg/m^2^ as obese [3]. The global incidence of overweight and obesity among adults has been observed to increase from 27.5% (857 million individuals) in 1980 to 47.1% (2.1 billion individuals) in 2013and 47.1%, respectively, between 1980 and 2013 [4]. Identifying and modifying early predictors of obesity can intervene, control obesity and thus reduce cardiovascular risk later in life [5, 6].

HTN, one of the major cardiovascular risk factors worldwide, is a widespread chronic condition with substantial death and disability rates. Although HTN incidence increases with age, recently, increasing attention has been given to the prevalence of HTN among young people. Globally, HTN prevalence in children has long been on the rise. Indeed, the global prevalence of HTN among children in 2014 reached 6.02%, representing a significant increase from a prevalence of 3.30% in the 2000s and 1.26% in the 1990s [7]. Numerous studies have shown that childhood HTN is associated with early-onset HTN and subclinical organ damage in adulthood, and that increased systolic blood pressure beginning in childhood is strongly associated with cardiovascular events before the age of 60 [8–11].

The relationship between HTN and obesity is complex, and some believe it to be “a 2-way street”, as HTN may lead through neuroendocrine processes and other mechanisms to increased BMI [12]. The complex mechanisms call for further epidemiologic studies. Our study utilized data from the Bogalusa Heart Study to primarily explore the associations between hypertension in non-obese children and adult BMI through regression analysis. Targeted prevention strategies for increase in early adult BMI based on the study findings are proposed to help improve public health.

## Methods

### Study cohort

Data for this study were obtained from the Bogalusa Heart Study in BioLINCC, a public medical database for Bogalusa, a semirural town in Louisiana in the USA. The Bogalusa Heart Study was designed to investigate risk factors for cardiovascular disease. Between 1973 and 1996, staff conducted a total of eight cross-sectional examinations, including seven cardiovascular risk factor screenings in children aged 5 to 17 years and one cross-sectional examination in adults(callde “Z510”). The current retrospective analysis consisted of participants who received anexamination during adulthood and at least one cross-sectional examination during childhood. Children measures in the current analysis were derived from the first cross-sectional examination in which children participated.

The Institutional Review Board of the Tulane University Health Science Center approved this study after informed consent was obtained from each participant or his or her legal guardian. The Medical Ethics Committee of Xiyuan Hospital, China Academy of Traditional Chinese Medicine approved the protocol for data analysis, and the National Heart Lung and Blood Institute (NHLBI) confirmed the study protocol and provided the data needed for the analysis.

**Fig. 1:**
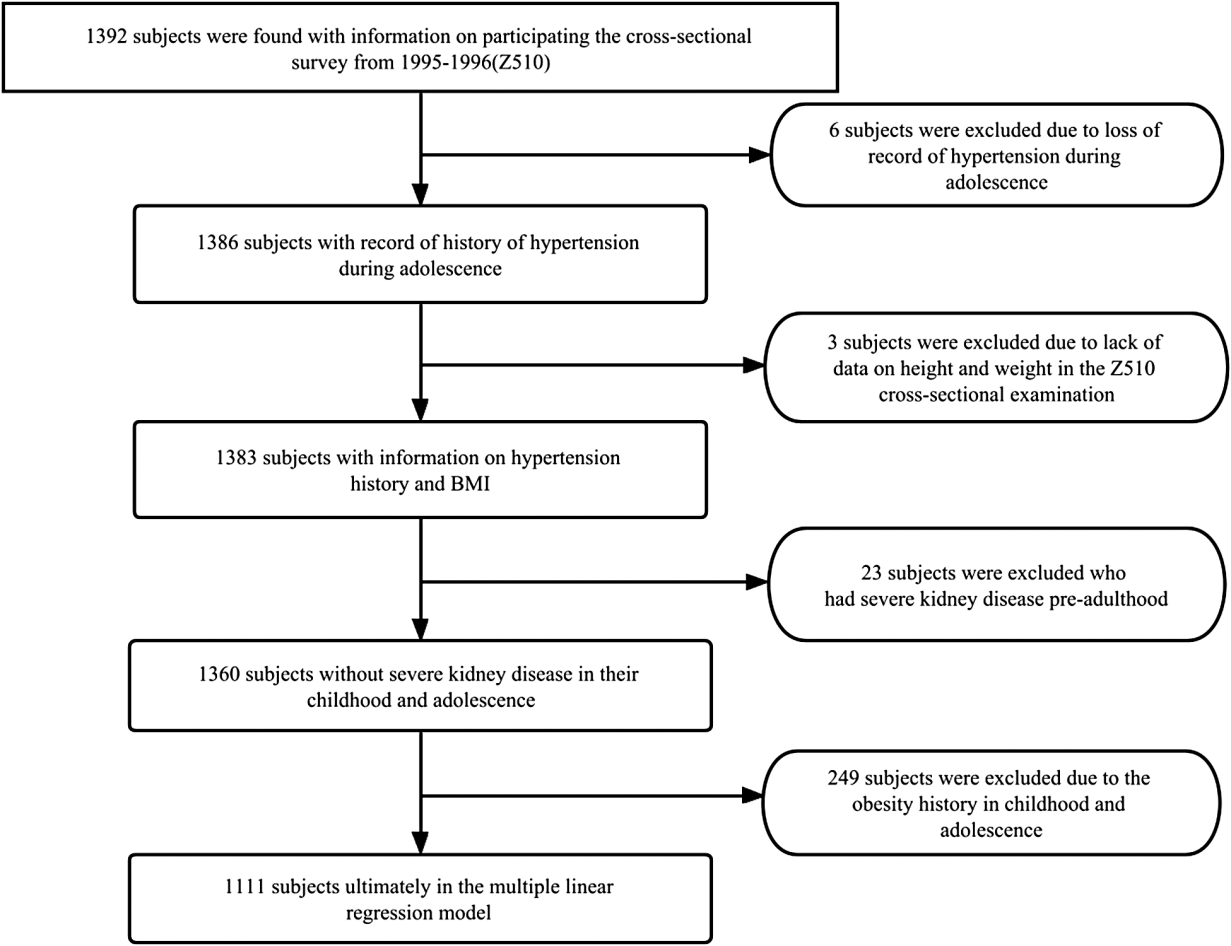
Flowchart of the study sample

### Measures

Data were collection by trained researchers following a standardized protocol. Participants were asked to fast for a minimum of eight hours before the examination. Health and medication histories were obtained by a questionnaire in a quiet and private setting. The questionnaire gathered information on the history of cardiovascular disease and other major diseases, smoking status and intake of alcohol, medication usage, etc. Nonsmokers were defined as those who had quit smoking for more than 6 months or smoked less than 1 cigarette per week. Nondrinkers were defined as those who had not consumed alcohol for more than 12 months. Blood pressure was measured in the right arm in a relaxed sitting position. Systolic and diastolic blood pressure measures were recorded using a mercury sphygmomanometer, with a mean of six repeated measurements was calculated. Blood samples were collected from the subject’s left arm by a registered nurse, with the right arm vein used in cases where blood could not be obtained from the left arm. Physical data were collected by trained examiners using specialized equipment and following standard procedures.

Hypertension history was determined by questionnaire responses given by the guardian and blood pressure data. During the childhood examination, nurses asked guardians if their child had high blood pressure. For the guardian responses that were uncertain or not recorded, blood pressure status was assessed according to the Fourth Report on the Diagnosis, Evaluation and Treatment of HTN in Children and Adolescents issued by the American Heart Association. For young children (i.e., 1–13 years old), “hypertension” was defined as a systolic and/or diastolic blood pressure ≥ 95th percentile based on age, sex, and height. For children > 13 years of age, elevated blood pressure and HTN were defined using the same criteria applied to adults, as outlined by the AHA and American College of Cardiology (ACC) [13]. Children with a BMI at or above the 95th percentile for age and sex were defined as obese according to clinical evaluation guidelines and the National Center for Health Statistics growth charts [14], and were excluded to avoid the indirect effects of obesity on blood pressure.

### Laboratory index analysis

Serum lipoprotein cholesterol and triglyceride levels were by heparin calcium precipitation and by agar gel electrophoresis using a Hitachi 902 automated analyser (Roche Diagnostics, Indianapolis, IN). Glucose levels were measured using an enzymatic procedure with a multichannel Olympus AU-5000 analyser (Olympus, Lake Success, NY). Plasma insulin levels were measured by a commercial radioimmunoassay kit (Padebas, Pharmacia Diagnostics, Piscataway, NJ).

### Statistical analysis

Statistical analysis was performed using R v.4.2.0 (the R Foundation; https://www.r-project.org/) and EmpowerStats software (R) (www.empowerststs.com; X & Y Solutions. Neneneba Inc.). The chi-square test and Kruskal-Wallis rank-sum test were used to compare the characteristics of hypertensive and nonhypertensive participants. A multiple linear regression model was used to determine the association between HTN in non-obese children and BMI in adulthood. Adult participants were then categorized into non-obese (BMI: 18-to < 25 kg/m^2^) and overweight/obese (BMI: ≥ 25 kg/m^2^) groups, logistic regression was used to assess the relationship between HTN in non-obese in children and BMI classification in adulthood. Demographic characteristics including sex, race, age, childhood measurements including fasting blood glucose (FBG) levels, serum insulin levels, pulse rate, high-density lipoprotein cholesterol (HDL-C) levels, low-density lipoprotein cholesterol (LDL-C) levels, total cholesterol (TC) levels, triglycerides (TG), and adult measurements including systolic blood pressure (SBP), diastolic blood pressure (DBP), history of coronary heart disease, asthma history, liver history, history of HTN medications, smoking history, and alcohol history were used as fixed effects, and an α value < 0.05 was considered to indicate a statistically significant difference. In addition, to examine potential interactions, participants were grouped according to race, FBG levels (< 126 mg/mL or ≥ 126 mg/mL), serum insulin levels (< 20 µU/mL or ≥ 20 µU/mL), SBP(< 120 mmHg or ≥ 120 mmHg), DBP(< 80 mmHg or ≥ 80 mmHg), LDL-C levels (< 130 mg/dL or ≥ 130 mg/dL), TC levels (< 200 mg/dL or ≥ 200 mg/dL), smoking history (smoker or non-smoker), and a history of alcohol intake (drinker or nondrinker). Generalized linear mixed models were used to test the interaction of these risk factors with HTN.

## Results

In this section, we discuss the association between hypertension in non-obese children and BMI in adulthood. A total of 1392 participants were initially included. After excluding individuals with missing blood pressure and physical data, individuals with obesity in childhood, and individuals with severe kidney disease in childhood to reduce the impact of secondary HTN. The final sample of the analysis included 1111 participants(38.97% males, 61.03% females) (Fig. 1), aged 20 to 37 years. Among the participants, 40 (3.60%) had HTN during childhood. During childhood, hypertension was more prevalent among black participants (6.69%-2.29%, *P* < 0.001). At the same time fasting blood glucose was significantly higher in non-obese children with hypertension at baseline (95.3–80.0, *P* < 0.001). As adults, 313 participants were overweight, and 331 were obese. Compared with participants with no history of HTN, participants with HTN in childhood had higher FBG levels (95.3–80.0, *P* < 0.001) and insulin levels (18.8–12.9, *P* = 0.002) in adulthood. Blood pressure was also significantly different between the two groups of participants, with increased systolic and diastolic blood pressure values in adults in participants that had HTN in childhood (SBP: 122.0-111.0, *P* < 0.001; DBP: 81.8–73.8, *P* < 0.001). There were no significant differences in sex, age, blood lipids, pulse rate, or smoking and drinking history between the two groups(Table 1).

**Table 1:** Characteristics of the study participants by hypertension status

Variables	Without HTN (1071)	With HTN (40)	Total	*P* value*
Age,y^b^	29.28 ± 5.13	29.85 ± 4.89		0.491
Sex			1111	0.609
Male, n (%)	417 (96.77%)	14 (3.23%)	431	
Female, n (%)	654 (96.18%)	26 (3.82%)	680	
Race			1111	< 0.001
White, n (%)	766 (97.71%)	18 (2.29%)	774	
Black, n (%)	305 (93.31%)	22 (6.69%)	327	
BMI, kg/m^2b^	27.4 ± 6.8	34.7 ± 9.5		< 0.001
BMI classification^b^			1111	< 0.001
BMI < 25 kg/m^2^, n (%)	462(98.93%)	5(1.07%)	431	
Overweight, n (%)	303(96.81)	10(3.19%)	313	
Obesity, n (%)	306(92.45%)	25(7.55%)	331	
HDL-C, mg/dL^a^	49.2 ± 13.9	46.6 ± 11.1		0.255
LDL-C, mg/dL^a^	12.9 ± 11.0	18.8 ± 19.3		0.857
TC, mg/dL^a^	189.8 ± 38.6	194.8 ± 47.9		0.430
TG, mg/dL^a^	112.4 ± 94.5	153.2 ± 281.0		0.912
FBG,mg/dL^a^	80.0 ± 11.0	95.3 ± 49.4		< 0.001
Insulin, µU/mL^a^	12.9 ± 11.0	18.8 ± 19.3		0.002
Pulse, beats per min^a^	71.8 ± 10.0	69.3 ± 14.2		0.126
DBP, mmHg^b^	73.8 ± 8.9	81.8 ± 11.2		< 0.001
SBP, mmHg^b^	111.0 ± 11.4	122.0 ± 14.0		< 0.001
Smoking status^b^			1043^	0.400
No, n(%)	624 (97.20%)	18 (2.80%)	642	
Yes, n(%)	386 (96.26%)	15 (3.74%)	401	
Drinking status^b^			1073^	0.218
No, (%)	751 (97.15%)	22 (2.85%)	773	
Yes, (%)	287 (95.67%)	13 (4.33%)	300	

* Kruskal‒Wallis test was used to assess for differences in continuous variables, and Fisher’s test was used for categorical variables. The chi-square test was used to compare characteristics between groups.

a: Measurements were obtained from the first cross-sectional examination performed during childhood; b:Measurements were obtained from the cross-sectional examination performed during adulthood.

^ The sample size used to calculate the statistics was smaller than the total sample size due to the lack of information on smoking status and alcohol consumption for some adult participants.

As shown in Table 2, after adjusting for race, FBG levels, serum insulin levels, and blood pressure based on Model 1, HTN in non-obese children was positively associated with BMI in adulthood (β = 2.64 kg/m^2^, 95% CI: 0.88–4.40, *P* = 0.0033). The correlation was not significantly altered by these adjustments. By stratified analysis, we found that in males with childhood HTN, BMI was 3.93 kg/m^2^ higher in adulthood compared to the female population (*P* = 0.0039).

Logistic regression was also used to calculate the association between childhood history of HTN and overweight or obesity in adulthood. As shown in Table 3, after full adjustment, participants with a history of childhood HTN were 3.71 times more likely to be overweight in adulthood than participants without a history of childhood HTN (95% CI: 1.11–12.46, *P* = 0.0337).

**Table 2:** Multivariate regression analysis of the effect of hypertension in non-obese children on BMI in adulthood

Models	β (95% CI)	*P* Value
Not adjusted	7.29 (5.11, 9.48)	< 0.0001
Model 1*	5.70 (3.67, 7.72)	< 0.0001
Fully adjusted	2.64 (0.88, 4.40)	0.0033

*Model 1 used a multiple linear regression model among participants with (n = 40) and without (n = 1071) hypertension during childhood. Age, sex, pulse rate, low-density lipoprotein cholesterol levels, high-density lipoprotein cholesterol levels, total cholesterol levels, history of heart disease, history of asthma, history of liver disease, medication for hypertension, smoking status and alcohol consumption were used as fixed effects.

**Table 3:** Logistic regression analysis of the effect of hypertension in non-obese children on overweight and obesity in adulthood

Models	OR (95% CI)	*P* Value
Not adjusted	5.01 (1.77, 14.18)	0.0024
Model 1*	5.23 (1.73, 15.77)	0.0033
Fully adjusted	3.71 (1.11, 12.46)	0.0337

We categorized measures according to those that were significantly different (Table 1) and prevalent cardiovascular risk factors such as LDL-C, to study the effect of HTN on BMI in different subgroups (Table 4). The observed interaction suggested a significant difference in BMI between the low and high LDL-C groups (0.73 vs. 4.80, *P* = 0.0245). Differences between the remaining subgroups were not statistically significant. Moreover, we found an interaction between alcohol consumption and HTN to be close to significant (*P* = 0.0504).

**Table 4:** Subgroup analysis of the effect of childhood hypertension on BMI

Subgroups*	BMI, kg/m^2^ (95% CI)	*P* Value	*P* Value for Interaction
Sex			0.9791
Male	2.61 (-0.30, 5.52)	0.0792	
Female	3.71 (1.53, 5.88)	0.0009	
Race			0.3718
White	3.47 (0.92, 6.02)	0.0078	
Black	3.82 (1.40, 6.23)	0.0020	
FBG, mg/mL			0.3007
< 126	3.32 (1.50, 5.15)	0.0004	
≥ 126	0.97 (-4.42, 6.36)	0.7237	
Insulin, µU/mL			0.1930
< 20	2.45 (0.49, 4.41)	0.0143	
≥ 20	11.78 (7.96, 15.60)	< 0.0001	
SBP, mmHg			0.6233
< 120	3.37 (0.88, 5.87)	0.0081	
≥ 120	4.54 (2.05, 7.03)	0.0004	
DBP, mmHg			0.8735
< 80	2.94 (0.59, 5.29)	0.0145	
≥ 80	4.16 (1.51, 6.82)	0.0022	
LDL-C, mg/dL			0.0245
< 130	0.73 (-1.60, 3.06)	0.5372	
≥ 130	4.80 (-6.04, 15.63)	0.3858	
TC, mg/dL			0.7407
< 200	2.91 (0.72, 5.10)	0.0092	
≥ 200	2.39 (-0.52, 5.29)	0.1074	
Smoking status			0.3303
Yes	1.52 (-1.23, 4.27)	0.2799	
No	4.40 (1.92, 6.89)	0.0005	
Drinking status			0.0504
Yes	4.36 (1.50, 7.22)	0.0029	
No	1.36 (-0.88, 3.59)	0.2339	

*All factors (age, race, sex, pulse rate, low-density lipoprotein cholesterol levels, high-density lipoprotein cholesterol levels, total cholesterol levels, triglyceride levels, blood pressure, FBG levels, insulin levels, history of heart disease, asthma, liver disease, medication for hypertension, smoking, alcohol consumption) were adjusted for in each stratification in addition to stratification factors.

## Discussion

In this retrospective observational analysis, we found a significant association between childhood HTN history and increased BMI in adulthood. The odds of being overweight or obese was 3.71 times higher in adults with a history of HTN as a child than that in participants without a history of HTN, with an average increase of 2.64 kg/m^2^. We also observed a significant interaction between HTN and LDL-C levels.

Obesity is an independent risk factor for cardiovascular disease [15], and the degree and duration of obesity can affect the occurrence and prognosis of cardiovascular disease. A high level of fat mass can cause dysregulation in cardiovascular risk factors such as blood lipids and blood glucose, while visceral fat promotes systemic and vascular inflammation, accelerates atherosclerotic plaque formation, and further increases the risk of cardiovascular events [16, 17]. Epidemiological studies have shown that 65–78% of the risk of essential HTN may be attributed to obesity [18]. The mechanism underlying HTN secondary to obesity is complex and mainly involves sympathetic nervous system activation, increased mineralocorticoid activity, sodium reabsorption, and insulin resistance [19]. Children with severe obesity have higher cardiovascular morbidity and all-cause mortality [5]. Exploring the relationship between hypertension in childhood and BMI in adulthood is important for preventing the development of cardiovascular disease and improving its prognosis. Previous studies have explored the correlation between hypertension and obesity in different populations. The Framingham study found an increased risk of developing obesity in adults with HTN over the age of 30 years, and the Tecumseh Blood Pressure Study found that patients with baseline HTN gained more weight than normal subjects and had significantly increased skinfold thickness [20, 21]. This study presents evidence that childhood HTN is strongly linked to adulthood obesity wherein children with HTN have a higher BMI in adulthood, thereby increasing the probability of becoming overweight and obese.

The mechanisms linking HTN to subsequent obesity are unclear, with possible explanations relating to sympathetic enhancement, renal injury, oxidative stress and changes in gut flora. Sympathetic tone is chronically increased in hypertensive environments, which leads to β-adrenergic downregulation and reduced calorie-burning capacity, while enhanced sympathetic tone stimulates eating activity, which contributes to weight gain. HTN is a disease involving the immune system and inflammation [22]. Under hypertensive conditions, immune responses and inflammation reinforce each other, forming the underlying pathophysiological processesy of HTN [23]. Oxidative stress is considered to be the unifying factor, and a strong association has been established with HTN [24, 25]. The development and progression of obesity has also been linked to an oxidative stress environment similar to HTN, in which the immune system shifts to a state of hyperinflammation, leading to the development of obesity-related diseases [26, 27]. Imbalances in the gut microbiota play a key role in the development and progression of HTN, which also significantly affects the structure and composition of the gut microbiota [28]. During the development of HTN, the abundance, diversity, and number of genes in the gut flora progressively decrease, and gut flora dysbiosis further aggravates HTN [29]. In hypertensive states, the relative abundance of bacteria in the gut is significantly lower, but the proportion of thick-walled bacterial phyla is significantly increased [30, 31]. The altered gut microbiota in obese patients is one of the reasons for the development of associated inflammation that is a hallmark of obesity and associated adverse outcomes [31, 32]. Alterations in the gut microbiota affect intestinal permeability, and activation of immune signalling pathways leads to a chronic low-grade inflammatory response, which is associated with an increased risk of obesity-related diseases. HTN and obesity often coexist, which induces metabolic disorders and inflammation, and further increase the burden on the cardiovascular system. The common pathological mechanism of the two diseases provides additional opportunities for treatment, such as with SGLT2 inhibitors, new treatment for diabetes, which have significant antihypertensive effects [33]. The same disease environment highlights the need for new class of drugs that target inflammation, which are effective in the treatment of HTN and obesity. In clinicalsetting, a better results can be achieved by targeting the common pathological pathway of the two diseases. An interaction between alcohol consumption and abdominal obesity on the increased risk of HTN has been found [34], but further research is requried to understand long-term drinking behaviour in hypertensive children increases the probability of becoming overweight or obese.

Among participants with childhood HTN, BMI was significantly higher in the subgroup with an LDL-C level ≥ 130 mg/mL, suggesting a potential interaction between HTN and LDL-C levels. A cohort study found that high BMI in dyslipidaemic groups was associated with increased cardiovascular risk [35], which suggests that hyperlipidaemic individuals that strictly control BMI and avoid becoming overweight and obesity in hyperlipidemic groups to reduce cardiovascular events.

There are some limitations in our study. First, BMI was used as the criterion to sole measure overweight and obesity, without considering the influence of body fat distribution. Second, the sample size of this cohort study was small, which could affect the robustness of the findings. Indeed the relationship between childhood HTN and obesity (BMI: ≥ 30 kg/m^2^) in adulthood using logistic regression, we did not get a significant difference; Third, data on HTN history were mainly obtained through questionnaires, which may have included incorrect responses, and the method in which blood pressure was measured in the cross-sectional examinations were unable for diagnosis. Fourth, the mechanism underlying the relationship between HTN and obesity was not investigated, which limits our understanding of causative effects.

## Conclusion

Overweight and obesity are considerable public health challenges worldwide. This study found that HTN in childhood was a predictor of overweight and obesity in adulthood. To reduce the risk of cardiovascular disease in the future, it is necessary to detect and control HTN as early as possible in children. In addition, families and society should work together to build healthier lifestyles throughout society by strengthening health education and preventive awareness to address the major public health challenge of obesity.

## Data Availability

The datasets generated and analysed during the current study are available in the BioLINCC, https://biolincc.nhlbi.nih.gov/home/.

## Abbreviations

BMI: body mass index
HTN: hypertension
DBP: diastolic blood pressure
FBG: fasting blood glucose
HDL-C: high-density lipoprotein cholesterol
LDL-C: low-density lipoprotein cholesterol
SBP: systolic blood pressure
SGLT2: sodium-glucose co-transporter-2
TC: total cholesterol
